# Cellulose-Based Interfacial Solar Steam Generation: Material Classification, Architectural Design, and Multifunctional Strategies

**DOI:** 10.3390/polym18131627

**Published:** 2026-06-30

**Authors:** Jiayuan Sun, Ling Jiang

**Affiliations:** 1 School of Water and Environment, Chang’an University, Xi’an 710054, China; jiayuan220766@163.com; 2 Key Laboratory of Subsurface Hydrology and Ecological Effect in Arid Region of the Ministry of Education, Chang’an University, Xi’an 710054, China; 3 Key Laboratory of Ecohydrology and Water Security in Arid and Semi-Arid Regions of Ministry of Water Resources, Chang’an University, Xi’an 710054, China

**Keywords:** interfacial solar steam generation, cellulose substrate, hierarchical structure, nanocellulose, desalination, sustainable water treatment, multifunctional design

## Abstract

The increasing global demand for freshwater, together with the high energy consumption and environmental footprint of conventional desalination technologies, has stimulated growing interest in interfacial solar steam generation (ISSG). ISSG is a solar-driven water purification strategy that localizes heat at the air–water evaporation interface, thereby promoting surface evaporation without heating the entire bulk water body. The development of efficient, durable, and multifunctional ISSG systems depends strongly on substrate materials that can regulate water transport, heat localization, vapor release, and mechanical stability. This review focuses on cellulose-based substrates for ISSG and examines how their molecular structure, fibrillar assembly, and macroscopic porous architecture influence evaporation behavior and device function. The reviewed cellulose platforms are classified into three major groups: bottom–up assembled nanocellulose substrates, including cellulose nanocrystals, cellulose nanofibers, and bacterial cellulose; natural hierarchical substrates, including wood, cotton fabrics, and agricultural residues; and commercial planar substrates, including cellulose paper and membranes. Beyond evaporation performance, this review discusses multifunctional design strategies for salt regulation, antifouling and antibacterial operation, water–electricity cogeneration, and photocatalytic pollutant degradation, with emphasis on their mechanisms and functional trade-offs. Finally, we identify critical bottlenecks limiting practical deployment and propose a roadmap for future intelligent, adaptive, and multi-energy-coupled cellulose-based ISSG systems. These systems offer a promising platform for distributed and resource-efficient water treatment, but their practical and environmental benefits depend on fabrication energy, material safety, device lifetime, and end-of-life management.

## 1. Introduction

**Figure 1:**
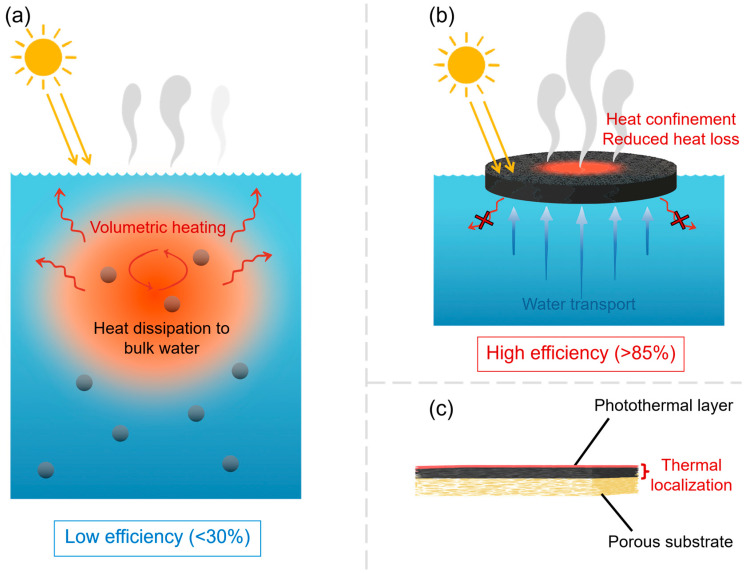
Comparison between (**a**) volumetric solar steam generation (VSSG) and (**b**) interfacial solar steam generation (ISSG). In ISSG, solar heat is localized at the air–water evaporation interface rather than distributed throughout the bulk water. (**c**) The schematic also shows the photothermal layer, cellulose-based transport substrate, water supply pathway, and vapor release region.

Rising global demand for freshwater exposes the sustainability limits of conventional seawater desalination. Techniques such as reverse osmosis (RO) and heat-driven multi-stage flash (MSF) or multi-effect distillation (MED) consume substantial energy, require complex infrastructure, and generate significant greenhouse gas emissions [1,2,3,4,5]. Interfacial solar steam generation (ISSG) addresses these issues by localizing solar heat at the air–water evaporation interface, where water is converted into vapor without heating the entire bulk water body. This approach can increase solar-to-steam (evaporation) efficiency from below 30% to over 85% [6,7,8,9,10], where the efficiency is defined as the fraction of incident solar energy converted into latent heat for vapor generation at the air–water interface. This improvement has made substrate and photothermal material design central to the further development of ISSG (Figure 1).

High-performance ISSG relies on both photothermal materials and porous substrates. Photothermal materials convert solar irradiation into localized heat, whereas substrates regulate water supply, vapor release, heat confinement, and mechanical support. Noble metal nanoparticles, such as Ag and Pd, harvest visible light through localized surface plasmon resonance [11,12,13,14], while semiconductors such as vanadium dioxide (VO_2_) provide broadband absorption and temperature adaptive photothermal response [15,16,17]. Carbon-based nanomaterials, including graphene and aerogels, provide durable and efficient photothermal layers [18,19,20]. Biomass- and polymer-derived carbons can reduce environmental burden and cost while retaining micro- and nanoscale architectures that facilitate light absorption and water transport [21,22].

Substrates that manage water supply, heat, and mechanical stability are equally crucial. Ideal substrates combine fast capillary transport, thermal localization, mechanical strength, and environmental compatibility [23,24,25,26,27,28,29]. Conventional polymer foams and hydrogels often fall short in hydrophilicity, integrity, or scalability. Cellulose-based substrates, with inherent hydrophilicity, hierarchical porosity, low thermal conductivity, mechanical robustness, and chemical modifiability, have emerged as important platforms for ISSG [30]. Rather than serving only as passive supports, cellulose-based substrates actively regulate capillary water supply, interfacial heat localization, vapor diffusion, salt redistribution, and interfacial interactions.

Most existing reviews organize biomass-derived evaporators, nanocellulose materials, wood-based generators, or multifunctional ISSG systems by material category or device function (Table 1). The fundamental design logic linking cellulose structure to transport regulation and device performance remains insufficiently clarified.

Cellulose is not merely a sustainable substrate for ISSG. Across different forms, including cellulose nanocrystals, cellulose nanofibers, bacterial cellulose, wood, textiles, agricultural residues, and paper-based materials, cellulose provides a hierarchical structural platform spanning molecular hydrogen-bond networks, fibrillar assemblies, interconnected pore systems, and macroscopic transport channels. These features, together with cellulose polymorphism across different allomorphs (I–V), govern water transport, interfacial hydration, heat localization, salt redistribution, mechanical stability, and multifunctional integration. Understanding how this structural hierarchy translates into functional performance is therefore essential for the rational design of next-generation ISSG systems.

This review is organized around a substrate structure–function–application framework rather than a simple inventory of cellulose materials. We first establish the structural basis of cellulose across multiple length scales and then examine how different cellulose architectures regulate mass transport, heat management, and interfacial processes. Subsequently, we discuss multifunctional ISSG systems, including salt-resistant, antifouling, water–electricity cogeneration, and photocatalytic platforms, with emphasis on functional trade-offs and coupling mechanisms. Finally, we evaluate challenges related to scalability, durability, sustainability, and practical deployment, and propose future directions toward adaptive, intelligent, and integrated cellulose-based water treatment systems. Sustainability is treated as a conditional attribute. Its environmental benefit depends on fabrication energy, drying method, nanomaterial use, device lifetime, and end-of-life management.

In this review, practical translation is assessed through fabrication scalability, process energy demand, material cost, durability under realistic water conditions, module compatibility, replacement feasibility, environmental safety, and indicative development stage rather than by evaporation rate alone.

## 2. Cellulose

### 2.1. Structure and Properties of Cellulose

**Figure 2:**
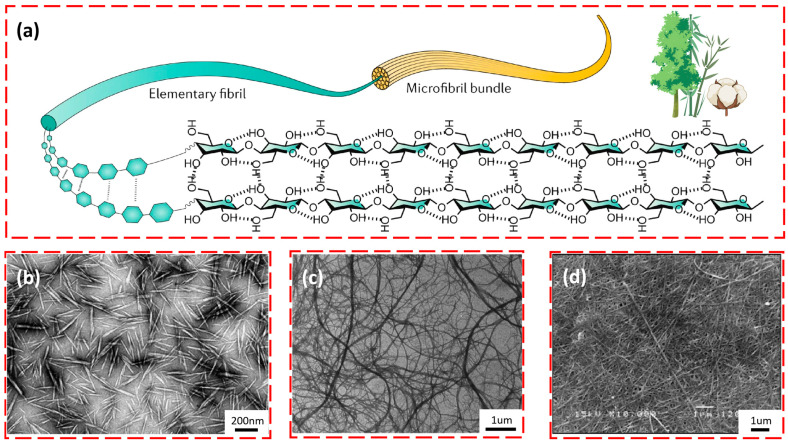
Hierarchical structure and representative morphologies of cellulose. (**a**) Schematic illustration of the hierarchical structure of cellulose. Reproduced from ref. [49] with permission from the copyright holder, © 2020, Springer Nature Limited. (**b**) TEM image of cellulose nanocrystals (CNCs). Reproduced from ref. [50]. © 2008 Royal Society Of Chemistry, (**c**) SEM image of cellulose nanofibers (CNFs). Reproduced from ref. [51]. © 1997 John Wiley & Sons, Inc. (**d**) SEM image of bacterial cellulose (BC). Reproduced from ref. [52] with permission from the copyright holder, © 2008 Elsevier Ltd. All rights reserved.

Cellulose is a linear polysaccharide composed of β-1,4-linked anhydroglucose units (AGUs). Hydroxyl groups at the C2, C3, and C6 positions form extensive intra- and intermolecular hydrogen-bonding networks (Figure 2a). These interactions reinforce the polymer backbone and give cellulose high axial stiffness [44,45]. The degree of polymerization (DP) varies with the source and processing. The DP of industrial cotton pulp ranges from 300 to 1170, whereas that of natural wood can approach 10,000, while BC often shows high DP and crystallinity, depending on biosynthesis conditions [46,47,48]. Chain length and structural regularity directly affect mechanical properties, processability, and operational stability.

At the supramolecular level, cellulose chains assemble into crystalline domains stabilized by hydrogen-bonding and van der Waals interactions, resulting in polymorphs from cellulose I to V [53,54,55]. These polymorphs regulate hydrogen-bond density, chain packing, and surface energy, directly influencing water adsorption, capillary transport, and interfacial evaporation in ISSG. Natural cellulose Iβ is dominant in higher plants, with monoclinic lattice and two-dimensional hydrogen bond network, which provides structural rigidity. Chemical treatment or regeneration converts cellulose I into cellulose II with anti-parallel chain stacking, while further treatment can produce cellulose III, IV and V under some conditions [56,57,58]. These polymorphic transformations provide a structural basis for regulating wettability, pore structure and surface energy.

Cellulose further assembles into hierarchical fibrillar structures. Basic fibrils formed by 18–24 cellulose chains are assembled into microfibers with a diameter of 10–30 nm through hydrogen bonding and hemicellulose-mediated interaction [49,59,60,61,62]. These fibrils assemble into larger fiber bundles that contribute to the hierarchical architecture of wood, cotton, and other plant-derived cellulose matrices. This multiscale structure transforms molecular rigidity into mechanical strength while creating interconnected paths for water transport and vapor diffusion.

On the macroscale, natural wood, cotton fabric and cellulose paper retain an obvious hierarchical network. Wood contains aligned ducts and tracheids for axial water transport, supported by nanoscale fiber networks within cell walls [63,64]. Cotton fiber has spiral fibril arrangement and surface grooves to enhance capillary action, while paper forms a three-dimensional porous network through interwoven micron-sized fibers [65,66]. In these matrices, multiscale porosity, inherent hydrophilicity, low thermal conductivity, mechanical integrity, and chemical modifiability provide the structural and physicochemical basis for cellulose-based ISSG systems [67,68]. The applicability of cellulose to ISSG depends on how its structure controls the coupling transportation process. Hydroxyl groups provide hydrophilicity and interfacial hydration. The fibrous network determines the continuity of pores and the supply of capillary water. Macro channels regulate vapor release and ion redistribution. These structural levels jointly affect evaporation rate, thermal constraint, salt tolerance, mechanical stability and long-term operation. Therefore, cellulose-based substrates should be regarded as active transport regulators rather than passive supports.

Polymorphic transformation of cellulose (I–V) is particularly related to ISSG properties because they determine the accessibility and bonding strength of hydroxyl groups in crystal domains. The conversion from natural cellulose I to regenerated cellulose II usually increases lattice disorder and weakens intermolecular hydrogen bonds, thus improving the accessibility of water and promoting the formation of intermediate water states that reduce the effective enthalpy of evaporation. In contrast, highly ordered crystalline regions limit swelling, but improve mechanical stability and directional capillary transport. Therefore, the phenomenon of cellulose polycrystal should be regarded not only as a structural classification, but also as a key regulating factor of water state evolution, interfacial thermal localization and evaporation kinetics in ISSG system.

### 2.2. Classification of Nanocellulose

Natural cellulose provides structural templates, but offers limited control over pore geometry, surface chemistry, and compositional distribution. Engineered nanocellulose broadens this design space because its morphology and surface functionality can be more precisely tailored. The three main forms are cellulose nanocrystals (CNCs) (Figure 2b), cellulose nanofibers (CNFs) (Figure 2c), and bacterial cellulose (BC) (Figure 2d). Their differences in rigidity, aspect ratio, network-forming ability, and surface chemistry define their roles in ISSG devices [34,69,70]. These three types of nanocellulose should not be regarded as interchangeable components. CNCs are more suitable for reinforcement and surface charge adjustment, because their rigid particles provide dimensional stability and charged interface. CNFs are more suitable for directional porous frames because their long fibers form a continuous network. BC is more suitable for moisture-stable membrane because biosyn-synthesis produces an interconnected nanofiber network, which does not contain lignin and hemicellulose. These differences explain why CNCs are often used as the modifier, CNFs as the aerogel framework, and BC as a self-supporting hydrogel or Janus membrane scaffold. In this paper, cellulose-based ISSG substrates are divided into three categories: self-assembled nanocellulose substrates, including CNCs, CNFs and BC; natural layered substrates, including wood, cotton fabrics and agricultural residues; and commercial planar substrate such as cellulose paper and cellulose film.

#### 2.2.1. Cellulose Nanocrystals

CNCs are rigid rod-shaped nanoparticles produced by acid hydrolysis, which selectively removes amorphous domains from natural cellulose [71,72]. Their high crystallinity and nanoscale dimensions provide structural stability, accessible surface area, and abundant sites for modification. Sulfuric acid hydrolysis introduces sulfate groups that improve water dispersibility and create charged interfaces for ion regulation. In ISSG devices, CNCs are used as reinforcing units, surface modifiers, and self-assembled porous components. They can also support photothermal modules or Janus structures to improve interfacial water management and salt tolerance [73,74,75].

#### 2.2.2. Cellulose Nanofibers

CNFs are flexible fibrils with high aspect ratios, usually obtained by mechanically disintegrating plant fibers after chemical or enzymatic pretreatment [76,77]. Their slender morphology promotes the formation of entanglement and network, making self-supporting hydrogels, aerogels and porous films possible. In ISSG device, CNFs are suitable for constructing directional channels and mechanically stable porous frames. Directional freezing, gradient freezing and related assembly methods adjust the orientation and connectivity of holes, allowing CNF-based structures to balance water supply, steam release and thermal positioning.

#### 2.2.3. Bacterial Cellulose

BC is biosynthesized by microorganisms as a pure nanofiber network free of lignin and hemicellulose [78,79]. This biosynthetic route enables the direct formation of hydrogel-like membranes with structural regularity and wet-state stability. Functionalization can be carried out during biosynthesis or after network formation by introducing graphene oxide, dopamine, metal nanoparticles, conductive polymers, or other photothermal components. In ISSG systems, BC serves as a scaffold for Janus membranes, vertically aligned aerogels, and multifunctional evaporators, supporting salt resistance, antibacterial activity, and stable operation in complex water environments.

## 3. Cellulose-Based Substrate Architectures for ISSG Devices

Previous studies have shown that cellulose-based porous networks can provide coupled pathways for water transport and light management in solar steam generators, highlighting the importance of substrate structure in ISSG design [80]. Therefore, this section discusses cellulose-based ISSG devices from the perspective of substrate architecture and transport regulation, with particular attention to how pore size, pore connectivity, channel orientation, fibrillar arrangement, wettability, and structural stability determine water supply, vapor release, heat confinement, salt redistribution, and long-term operation.

### 3.1. Bottom–Up Assembled Nanocellulose Substrates

The nanocellulose structure assembled from the bottom to the top mainly uses CNFs and BC to construct aerogels, hydrogels and porous membranes. Different from natural cellulose matrix, these systems can control pore geometry, component distribution and interface function more closely. Their design has changed from a simple porous scaffold to a multifunctional ISSG device, which integrates evaporation with salt management, pollutant degradation, antibacterial activity, energy storage or solar chemical conversion. The bottom–up nanocellulose structure provides precise structural control, but the processing cost is high and drying leads to shrinkage. Therefore, their performance should be judged by whether the designed pores remain open during wet operation, whether the heat is confined near the evaporation surface, and whether the network can resist shrinkage during repeated hydration.

#### 3.1.1. CNF-Based Aerogel

CNF-based aerogels are commonly prepared through physical entanglement, chemical crosslinking, directional freezing, or template-assisted assembly (Table 2). Their interconnected fibrillar networks provide structural continuity, while freezing-induced ice growth can generate aligned pores for liquid transport and vapor release [81]. Current CNF aerogel designs can be grouped into three directions, namely anisotropic transport control, interfacial water state regulation, and scalable multifunctional integration.

Anisotropic channel engineering is used to coordinate mass transfer, heat flow, and mechanical stability. Beam–column networks constructed from carbon nanofibers, carbon nanotubes, and MXene (two-dimensional transition metal carbides/nitrides) improve compressive recovery and maintain pore integrity during wet operation [82]. Bidirectional freezing creates vertical and radial pathways for axial water supply and lateral replenishment, reducing heat dissipation [83]. Janus CNF MXene aerogels introduce asymmetric wettability, allowing water transport and surface evaporation to occur in spatially separated regions. By separating water uptake from surface evaporation, this asymmetric configuration reduces salt accumulation near the photothermal surface and improves interfacial stability [84].

Interfacial chemistry provides another route to regulate evaporation behavior. Carboxylated CNF hydrogels combined with photothermal carbon layers can strengthen cellulose–water interactions and reduce the apparent evaporation enthalpy or alter the interfacial water state [85]. Bilayer aerogels further separate photothermal conversion, water transport, and airflow-assisted energy exchange, allowing for ISSG operation beyond passive daytime evaporation [86]. Similar bilayer structures using graphene oxide or related photothermal layers spatially decouple heat generation from brine transport, which helps suppress salt crystallization at the evaporation interface [87].

Scalable manufacturing has become increasingly important as CNF aerogels move from laboratory structures to device-oriented platforms. Foam skeleton templating enables CNF graphene aerogels to be dried under ambient conditions, reducing dependence on energy-intensive freeze-drying [88]. Functional CNF aerogels that combine photothermal evaporation with photocatalysis or peroxide generation expand ISSG from freshwater production to water treatment and solar chemical conversion [89]. Evaporation enhancement depends on fibrillar network coordination of water supply, vapor diffusion, heat retention, and pore stability—not on the photothermal component alone. The aligned pores shorten the water transport path and promote steam escape, while Janus wettability spatially separates the water absorption from the surface evaporation, thus reducing the salt retention near the heating area. Crosslinking or a beam–column network maintains pore integrity during wet compression, which explains why mechanically reinforced CNF aerogels are more stable during repeated hydration. However, the same structural accuracy often depends on directional freezing or freeze-drying, which improves the transportation rules but limits the readiness of large-scale production and manufacturing. Evaporation performance is strongly influenced by light intensity, feed water composition, salinity, ambient temperature, humidity, airflow and absorber geometry. Therefore, unless the test conditions are normalized, direct comparison between different studies should be interpreted with caution.

**Table 2:** CNF-based hybrid aerogel evaporators: condensed benchmarking, mechanistic roles, and limitations.

Material	Substrate	Fabrication Strategy	Testing Context	Representative Performance	Function	Structure-Controlled Role	Trade-Off/Limitation	Reference
CNF/PEI@CNT/MXene aerogel	CNF aerogel	Directional freeze-drying; amine crosslinking	1 sun; real seawater	2.02 kg m^−2^ h^−1^; 99.88%	Desalination; anti-scaling	Beam–column pores stabilize water channels	Freeze-drying; MXene stability	[82]
NOGF/CNF aerogel	CNF aerogel	Bidirectional freeze-casting	Condition NR	2.39 kg m^−2^ h^−1^	Stable evaporation	Vertical/radial channels guide transport	Freezing-dependent pore alignment	[83]
MXene/CNF Janus aerogel	CNF aerogel	Unidirectional freezing; Janus assembly	1 sun; salinity NR	2.29 kg m^−2^ h^−1^; 88.20%	Salt resistance	Janus wettability separates uptake/evaporation	MXene oxidation	[84]
CuS@CNF/PVA aerogel	CNF/PVA aerogel	Crosslinking; CuS coating	Condition NR	3.20 kg m^−2^ h^−1^	Dye degradation; salt resistance	Hydratable Janus skeleton supports coupled purification	Catalyst loading may block pores	[90]
HZC–CNF Janus aerogel	TEMPO-CNF aerogel	TEMPO oxidation; Janus construction	Condition NR	1.97 kg m^−2^ h^−1^; 92.80%	Pollutant removal	Vertical channels and conical roof guide vapor/solute transport	Complex architecture	[91]

Note: For Table 2, Table 3, Table 4, Table 5 and Table 6, the listed evaporation rates and efficiencies are representative values under different testing conditions and should not be used for direct ranking. The comparison focuses on the dominant structure-controlled role and the associated trade-off or limitation of each substrate design.

#### 3.1.2. BC-Based Aerogels and Hydrogels

BC is biosynthesized directly as a nanofiber hydrogel network, which simplifies scaffold formation compared with plant-derived CNFs [92,93]. Its functionalization generally follows two routes. In situ integration introduces photothermal precursors or nanoparticles during biosynthesis, allowing them to become embedded in the growing BC network. Post synthesis modification loads functional components after network formation and provides broader material compatibility [94,95].

Early BC-based ISSG systems established photothermal activity by incorporating polydopamine, black TiO_2_, silver nanoparticles, or polypyrrole into BC networks [33,94,95,96] These studies showed that BC can support evaporation and antibacterial functions without extensive structural reconstruction. Later designs shifted toward bioinspired organization and anisotropic transport. Aerosol-assisted biosynthesis produced multilayer evaporators that integrate photothermal conversion, water transport, and thermal insulation within one continuous structure [97]. Gradient freezing generated vertically aligned BC aerogels, while MXene incorporation enhanced solar absorption and improved operation in brine, wastewater, and acidic media [98].

**Table 3:** BC-based photothermal evaporators: condensed benchmarking, mechanistic roles, and limitations.

Photothermal Material	Substrate	Fabrication Strategy	Testing Context	Representative Performance	Function	Structure-Controlled Role	Trade-Off/Limitation	Reference
VTMS-modified Janus BC aerogel	BC network	Silane-regulated Janus assembly	Outdoor test reported	1.91; 4.20 kg m^−2^ h^−1^ outdoor	Salt resistance	Wetting contrast controls water/salt transport	Large-area Janus control	[92]
Ag NPs/PPy	BC	In situ reduction/polymerization	Condition NR	1.49 kg m^−2^ h^−1^	Antibacterial; flexible	Ag adds antibacterial function; PPy heats interface	Metal leaching risk	[94]
Polydopamine (PDA)	BC	In situ growth	1 sun; water type NR	1.13 kg m^−2^ h^−1^	Biodegradable evaporation	PDA provides photothermal conversion	Modest evaporation rate	[33]
Black TiO_2_	BC	Nanoparticle embedding	Real-time condition; salinity NR	1.26 kg m^−2^ h^−1^	Desalination; purification	Black TiO_2_ improves absorption; BC transports water	Particle retention unclear	[96]
MXene	BC aerogel	Gradient freezing	Brine/wastewater/acid media reported	1.78; 3.43 kg m^−2^ h^−1^	Desalination; acid stability	Aligned BC channels plus MXene heating	MXene oxidation; drying shrinkage	[98]
CNTs/BC nanocomposite	BC-based wood substrate	Aerosol-assisted biosynthesis	Condition NR	2.90 kg m^−2^ h^−1^	Reduced vaporization enthalpy	Multilayer structure couples transport and enthalpy reduction	Scale uniformity	[97]

Recent BC architectures place greater emphasis on energy management and interfacial patterning. Phase-change BC composites store solar heat and sustain evaporation under dark conditions [99]. Janus BC aerogels prepared from a homogeneous precursor system create superhydrophilic and superhydrophobic regions within a continuous network, improving water management under outdoor conditions [92]. These developments have positioned BC as a biosynthetic platform of ISSG system with moisture stability and adjustable structure, especially in the case of salt resistance, antibacterial operation and day-and-night evaporation. However, BC-based systems still face the challenges of scalable biosynthesis, thickness control, drying-induced shrinkage and uniform functionalization across large areas of membranes. Compared with CNF aerogels, BC provides better wet structural continuity, because its network is formed during biosynthesis, rather than after assembly. This feature is beneficial to stable water transport and repeated hydration. Its disadvantage is that the geometric programmability is weak because the pore size, membrane thickness and composition distribution are difficult to adjust over a large area. Therefore, the performance difference between BC-based evaporators mainly comes from the degree of hole arrangement, the contrast of interfacial wettability, the distribution of photothermal components and the ability of biosynthetic network to remain open during repeated wet operation.

**Table 4:** Wood-based photothermal evaporators: condensed benchmarking, mechanistic roles, and limitations.

Photothermal Material	Substrate	Fabrication Strategy	Testing Context	Representative Performance	Function	Structure-Controlled Role	Trade-Off/Limitation	Reference
Bamboo carbon black	Sulfonated wood	Delignification; sulfonation	Condition NR	3.40 kg m^−2^ h^−1^	Low enthalpy evaporation	Sulfonated channels enhance hydration and capillary supply	Dimensional stability	[100]
EHL-PANI coating	Balsa wood	Surface coating	Condition NR	2.10 kg m^−2^ h^−1^	Desalination; wastewater treatment	Lignin-PANI coating improves absorption	Coating uniformity	[101]
Furfurylated wood	Various wood	Surface furfurylation	Condition NR	1.64 kg m^−2^ h^−1^	Salinity adaptability	Polymer layer improves stability and ion purification	Reduced biodegradability	[102]
PPy/AgNPs wood	Delignified wood	PPy/Ag growth	Condition NR	2.04 kg m^−2^ h^−1^	Salt resistance; cogeneration	Vertical channels plus photothermal/antibacterial layer	Ag leaching; coating stability	[103]
WO_3−x_ nanorods	Wood	Surface nanorod loading	1 sun reported	82.50% efficiency	Broadband absorption	Nanorods enhance light trapping; wood supplies water	Nanorod adhesion	[104]
CB/MOF-801	Pine wood	In situ growth/loading	Condition NR	2.54 kg m^−2^ h^−1^	Desalination; wastewater treatment	Arched geometry increases evaporation area	MOF stability; shape control	[105]
Graphene flake/PANI	Wood sponge	GF/PANI coating	Condition NR	1.49 kg m^−2^ h^−1^	Salt resistance; self-cleaning	Conductive porous coating improves heating	Heat leakage if overcoated	[106]
Spiropyran-modified wood	Wood	Photoresponsive modification	Condition NR	NR	Dynamic salt discharge	Light-responsive wettability regulates salt removal	Chemical fatigue	[107]
MXene/PDES wood	Delignified wood	Impregnation-polymerization	Condition NR	1.59 kg m^−2^ h^−1^	Water-electricity cogeneration	Flexible wood channels support heat recovery	MXene aging	[108]
PPy-DES wood	DES-delignified wood	DES treatment; PPy polymerization	Condition NR	1.94 kg m^−2^ h^−1^	Salt self-cleaning	DES improves hydrophilicity and salt transport	Wood-type dependence	[109]
Ni-P/graphite wood	Wood	Electroless plating; graphite coating	1 sun + 2 V	2.20 kg m^−2^ h^−1^	Round-the-clock operation	Joule heating enables dark operation	External energy input	[110]
Chinese ink	Drilled wood sheet	Ink coating; drilling	Condition NR	1.60 kg m^−2^ h^−1^	Salt rejection; antifouling	Drilled pathways guide salt discharge	Manual channel design	[111]
Polythiophene	Fir veneer	Vapor deposition growth	Condition NR	1.42; 1.48 kg m^−2^ h^−1^	Salt resistance; wastewater treatment	Ultrathin veneer shortens transport distance	Coating durability	[112]

**Table 5:** Fabric-based photothermal evaporators: condensed benchmarking, mechanistic roles, and limitations.

Photothermal Material	Substrate	Fabrication Strategy	Testing Context	Representative Performance	Function	Structure-Controlled Role	Trade-Off/Limitation	Reference
Cotton/PPy/CNC fabric	Cotton fabric	PPy polymerization; CNC modification	1 sun; simulated/oily seawater	2.02; 1.90 kg m^−2^ h^−1^; 93.80%	Antifouling; oil resistance	PPy heats; CNC hydrates and resists oil	Washing/coating durability	[113]
Waste-cotton Janus evaporator	Waste-cotton fabric	Micro/nano Janus construction	Condition NR	3.16 kg m^−2^ h^−1^	Salt resistance	Wetting asymmetry separates supply and evaporation	Waste-textile variability	[114]
rGO-coated cotton fabric + TEG	Cotton fabric	rGO coating; TEG integration	1 sun; seawater/brine reported	1.39 kg m^−2^ h^−1^; 86.98%	Freshwater-electricity-H_2_	rGO heats; TEG recovers waste heat	Device complexity	[115]
CB@CA/CF wicking fabric	Cotton fabric	Breath-figure templating	1 sun; pure water/3.5 wt% NaCl; 8 h salt test	2.08; 1.98 kg m^−2^ h^−1^	Salt rejection	Porous CB@CA layer plus cotton wicking	Pore uniformity	[116]
Twisted CNT/cotton yarn fabric	Cotton/CNT yarn	Helical twisting	Condition NR	2.83 kg m^−2^ h^−1^	Salt resistance	Twisted yarns create coupled heat/water pathways	CNT cost; yarn uniformity	[117]

### 3.2. Natural Hierarchical Cellulose Substrates

Natural-graded cellulose substrates use pre-formed biological structures to construct ISSG devices with limited structural reconstruction. Wood, cotton fabric and agricultural residues are different in morphology and machinability, but their design follows a common logic. Photothermal, interfacial and catalytic components are retained, recombined or combined to improve evaporation, salt regulation, environmental stability and equipment integration. Natural cellulose matrices are different from nanocellulose aerogels because their transport paths are hereditary, not completely designed. This leads them to have lower manufacturing complexity and better structural integrity, but it also brings variability in pore size, channel orientation and chemical composition. Therefore, their performance is controlled by how much natural hierarchical structure is retained and how to uniformly introduce photothermal or interface functions.

#### 3.2.1. Wood Substrates

Wood-based ISSG systems mainly follow two design routes. The first route modifies the internal structure and chemistry of wood to regulate water state and transport. Delignification, sulfonation, and related treatments alter cellulose water interactions within aligned channels, reduce evaporation enthalpy, and improve interfacial evaporation [100]. By modifying the substrate rather than simply adding a surface absorber, this route links wood chemistry with evaporation thermodynamics.

The second route functionalizes the wood surface with photothermal or conductive layers. Lignin-derived polyaniline coatings, furfuryl carbon layers, and in situ polymerized polypyrrole- or silver-containing coatings enhance solar absorption while preserving the underlying wood framework [101,102,103]. Microstructural engineering, including drilled channels, arched geometries, and MOF decoration, expands the effective evaporation area, guides brine transport, and connects macroscopic shape design with interfacial water regulation [104,105].

Recent wood-based systems have moved from evaporation enhancement toward operational stability and functional coupling. Hydrophobic or conductive coatings improve salt tolerance and self-cleaning behavior [106], while photoresponsive spiropyran layers enable light-controlled wettability switching for salt discharge [107]. Flexible wood composites integrated with MXene, deep eutectic solvents, thermoelectric modules, or Joule heating layers extend wood evaporators to cogeneration, wastewater purification, and continuous operation [108,109,110]. Wood therefore functions not only as a passive biological template, but also as a chemically and geometrically reconfigurable device support. Its main limitations arise from structural variability, anisotropic swelling, and the difficulty of achieving uniform chemical modification across thick or irregular substrates. Wood-based ISSG performance depends on preserving axial transport channels, not only on photothermal coating. The remaining lumens and tracheids provide low-resistance water supply and allow salt to redistribute along the growth direction. Surface photothermal coating improves light absorption, but too much coating will block the lumen and weaken capillary flow. Chemical treatments such as delignification and sulfonation can increase hydrophilicity and change evaporation enthalpy, but they may also reduce dimensional stability. Therefore, wood-based ISSG should balance channel protection, surface absorption and swelling resistance, rather than maximizing coating loading alone.

#### 3.2.2. Cotton Fabric Substrates

Cotton fabrics support flexible ISSG devices through textile-compatible processes, including surface polymerization, hydrothermal growth, coating, weaving, and yarn engineering [113,114,115]. Their main advantage lies in scalable processing, mechanical flexibility, and compatibility with continuous textile manufacturing rather than high-precision structural control.

Cotton-based ISSG design can be divided into surface photothermal engineering, asymmetric wetting design, and textile-scale structural regulation. Surface photothermal engineering deposits polypyrrole, MoS_2_, reduced graphene oxide, carbon black, or related absorbers onto cotton fibers to improve solar absorption and interfacial heating [113,114,115]. When combined with photothermal polymer coatings, CNCs can further regulate hydration layers and improve oil resistance [113]. Asymmetric and Janus structures separate light absorption, water transport, and salt exclusion within one textile. All-cellulose wicking fabrics and waste-cotton Janus evaporators use directional water supply and wetting gradients to suppress salt deposition under saline or oily conditions [116].

Yarn and fabric scale engineering further shifts cotton-based ISSG from coating modification to textile architecture design. Helically twisted photothermal yarns, asymmetric hybrid fabrics, and multilayer braided structures coordinate water transport, thermal localization, and vapor release [117,118]. Cotton-based systems are also being developed as energy-coupled devices. Asymmetric photothermal fabrics can support simultaneous evaporation and electrical output [119], while reduced-graphene-oxide-coated cotton fabrics integrated with thermoelectric modules and electrolyzers connect desalination, waste-heat recovery, and hydrogen production in one system [115]. These features make cotton fabrics more suitable for flexible, scalable, and multifunctional ISSG modules than rigid laboratory scale evaporators. Their long-term performance, however, depends on coating adhesion, washing stability, mechanical fatigue resistance, and fouling control during repeated wet operation. Cotton-fabric evaporator performance depends on textile-scale water pathways and coating durability. Fiber weaving provides flexible capillary networks and mechanical compliance, while surface coating supplies photothermal conversion and functional wettability. However, repeated bending, washing, and salt exposure can detach coatings, alter wettability, and reduce long-term evaporation stability. Cotton-based ISSG is therefore more suitable for flexible and scalable modules, but its practical performance depends on interfacial bonding between cellulose fibers and functional layers rather than on the initial evaporation rate alone.

#### 3.2.3. Agricultural Waste Fiber Substrates

Agricultural residues, including rice husk, bagasse, corn straw, crop straw, and animal manure, provide low-cost feedstocks for cellulose-based ISSG [120,121]. Their relevance comes from the combination of waste valorization and inherited or reconstructed porous structures. Compared with wood and cotton, this route places greater emphasis on resource recycling, low processing cost, and feedstock diversity.

Direct carbonization preserves native biological channels while generating photothermal carbon frameworks. Carbonized corn straw, for example, retains anisotropic vascular bundles, and its cutting direction and channel arrangement affect water transport [122]. Direct carbonization minimizes processing, but provides limited control over pore hierarchy and compositional distribution. More controlled strategies deconstruct biomass into cellulose or nanocellulose and then rebuild ordered aerogels or composite networks. Ionic-liquid-assisted sol gel assembly of straw-derived microcrystalline cellulose produces anisotropic channels with low tortuosity [123], while straw-fiber-reduced graphene oxide aerogels combine biomass scaffolds with photothermal components [124].

Full component utilization offers a more sustainable design route. Bagasse can be separated into carbonized fractions for light absorption and CNF fractions for structural reconstruction, converting one waste stream into a composite aerogel [32,125]. Integration of tin with bagasse-derived nanocellulose produces superhydrophobic and elastic aerogels with improved environmental adaptability [126]. Biochar derived from animal manure further expands the feedstock range beyond plant residues and shows that nontraditional agricultural wastes can serve as precursors for photothermal evaporators [127]. These studies shift biomass-derived ISSG from simple carbonized absorbers toward reconstructed, multifunctional, and resource-efficient materials. The main barriers are feedstock heterogeneity, batch-to-batch variability, and the need to balance low-cost processing with reproducible pore structure and photothermal performance. Agricultural-residue evaporator performance depends on whether native or reconstructed channels survive processing, not solely on feedstock cost. Their value depends on whether native vascular channels or reconstructed cellulose networks can be retained after processing. Direct carbonization is simple and preserves part of the biological architecture, but it provides limited control over pore size, surface chemistry, and batch reproducibility. Reassembly routes offer better structural control and more predictable water transport, but they increase the processing cost and weaken the low-cost advantage of waste-derived substrates. The practical choice therefore depends on whether the target application prioritizes low-cost water production or reproducible device performance.

### 3.3. Commercial Planar Cellulose Substrates

Commercial planar cellulose substrates, including filter paper, cellulose paper and dust-free paper, represent the standardized ISSG platform. Unlike wood, cotton fabrics or nanocellulose aerogels, these substrates do not rely on complex biological levels or complex bottom–up assembly. Their value lies in their low cost, easy replacement and compatibility with coating, filtration, carbonization, lamination and continuous processing. Therefore, the paper-based substrate can not only be used as a model system for the study of interfacial evaporation, but also as a practical candidate for the expandable evaporator module.

Cellulose paper and dust-free paper are planar porous materials assembled by cellulose fibers, but they are different in manufacturing route and structural uniformity. Filter paper is usually produced by wet papermaking and contains interwoven fibers with relatively uniform pores. Dust-free paper is usually made by an air-laid process, and usually shows looser fiber accumulation and higher porosity. These differences will affect the diffusion of water, the release of steam and the loading of functional components. However, in the design of ISSG, both substrates provide low-cost porous substrates, which can be transformed into photothermal films by direct carbonization or surface functionalization.

Early studies confirmed the feasibility of using commercial paper products as solar evaporator precursors. Lin et al. [128] converted cellulose paper and related fiber products into free-standing porous carbonized membranes through one-step carbonization. This treatment transformed the original paper network into a lightweight photothermal structure while retaining porosity for water transport and vapor release. The importance of this work lies not in record evaporation performance, but in demonstrating that widely available paper can be upgraded into a self-supporting evaporator without expensive nanomaterials or complex equipment.

Subsequent paper-based ISSG research shifted toward nanomaterial integration. Conductive or photothermal nanosheets can be incorporated into cellulose-derived membranes to improve light absorption and extend device function. For example, ZnO modified MXene combined with CNFs produces a composite membrane that couples interfacial evaporation with thermoelectric output [72]. In this structure, the cellulose framework supports film formation and water management, while MXene provides photothermal and electrical functions. The limited growth of silver nanoparticles in the micron gap of cellulose paper provides another way to enhance broadband light absorption through plasma effect and close interface contact [129].

**Table 6:** Cellulose-based photothermal paper evaporators: condensed benchmarking, mechanistic roles, and limitations.

Photothermal Material	Substrate	Fabrication Strategy	Testing Context	Representative Performance	Function	Structure-Controlled Role	Trade-Off/Limitation	Reference
CNF@ZNM-MXene film	CNF	Vacuum filtration; ZnO-modified MXene	Condition NR	1.27 kg m^−2^ h^−1^; 82.15%	Water-electricity cogeneration	CNF supports film; MXene heats and conducts	MXene oxidation	[72]
Carbonized cellulose paper	Commercial cellulose paper	One-step carbonization	1 sun; pure water	0.96 kg m^−2^ h^−1^; 65.80%	Performance benchmark	Carbonized paper forms low-cost photothermal network	Moderate efficiency	[128]
Cellulose-based carbon paper	Biomass-derived paper	Freeze-drying; carbonization	Seawater/wastewater reported	1.25 kg m^−2^ h^−1^; 80.60%	Salt rejection; purification	Hierarchical carbon paper supports flexible evaporation	Drying/carbonization energy	[130]
Ag-CP plasmonic paper	Cellulose paper	Confined Ag synthesis	Condition NR	85.20% efficiency	Broadband absorption	Ag nanoconfinement enhances plasmonic heating	Ag cost/leaching	[131]
HZC–CNF Janus aerogel	TEMPO-CNF aerogel	Directional freezing; Janus design	Condition NR	1.97 kg m^−2^ h^−1^; 92.80%	Purification coupling	Vertical channels guide water and pollutant contact	Closer to aerogel than paper	[91]

Structural reconfiguration has also become important for paper-based evaporators. TEMPO oxidized CNFs followed by directional freezing and carbonization can reorganize cellulose fibers into anisotropic or Janus like porous structures [91]. The reconfiguration of this structure improves the directional transportation of water and steam diffusion, and allows pollutants to be adsorbed or degraded in the same evaporator. Biomass-derived photothermal paper prepared by freeze-drying and carbonization further shows that pore connectivity can be adjusted to support salt diffusion and long-term seawater operation [130]. In these systems, cellulose paper is no longer treated as a simple flat support, but as a processable precursor for hierarchical porous membranes.

Therefore, the development of commercial planar cellulose substrate can be understood as the progress from direct carbonization to functional loading and structural reconstruction. Direct carbonization makes paper a low-cost photothermal precursor. Nano-material integration improves solar energy absorption and expands equipment functions. Oxidation, freeze-drying, carbonization and Janus design further adjust the pore structure, salt tolerance, pollutant removal and operational stability. Compared with natural wood and cotton fabric, commercial paper substrate has weaker intrinsic anisotropy and simpler hierarchical structure. Compared with nanocellulose aerogels, their structural accuracy is lower. On the contrary, their advantages lie in manufacturability, standardization, substitutability and compatibility with large-scale processing. These characteristics make them suitable for a replaceable evaporator layer, a disposable water treatment module and an upgradeable ISSG prototype. Paper-based substrates should be judged by standardization and replaceability, not by peak evaporation rate. Their weak intrinsic anisotropy limits directional water regulation, but their structural uniformity supports reproducible coating, lamination, filtration, and carbonization. This makes paper less suitable for highly programmed salt migration than wood or aligned aerogels, but more suitable for disposable modules, scalable prototypes, and low-cost performance screening where reproducibility and replacement are more important than structural sophistication.

The studies summarized above indicate that porous cellulose substrates should be compared through structure-dependent transport and energy management principles rather than by evaporation rate alone.

### 3.4. Fundamental Principles for Selecting Porous Cellulose Substrates

Section 3.1, Section 3.2 and Section 3.3 indicate that porous cellulose substrates should be selected by transport and energy management principles, not by evaporation rate alone. An effective substrate must simultaneously support continuous capillary water supply, rapid steam release, interfacial thermal localization, salt redistribution and mechanical stability under wet operating conditions. Therefore, when selecting the cellulose platform, pore size, pore connectivity, channel orientation, surface wettability, thermal conductivity and structural robustness should be considered comprehensively.

Different cellulose substrates meet these requirements in different ways. CNF aerogels provide the highest structural adjustability, because their pore orientation, network density and wettability can be programmed during the assembly process, making them suitable for machinery-oriented and high-performance equipment, although many systems still rely on high-energy drying. BC aerogels and hydrogels provide wet-stable nanofiber networks, but large-scale biosynthesis and thickness control are still difficult. When the aligned channels are retained, the wood matrix is beneficial because these channels support axial water supply and salt redistribution, but natural heterogeneity affects reproducibility. Cotton fabric provides flexibility and textile scale processing, but the coating adhesion and washing stability determine long-term use. Agricultural resources reduce the cost of materials and contribute to the reuse of wastes, while the diversity of raw materials complicates quality control. Commercial cellulose paper provides standardization and easy replacement, but its weak intrinsic anisotropy limits the advanced directional transmission rules.

**Figure 3:**
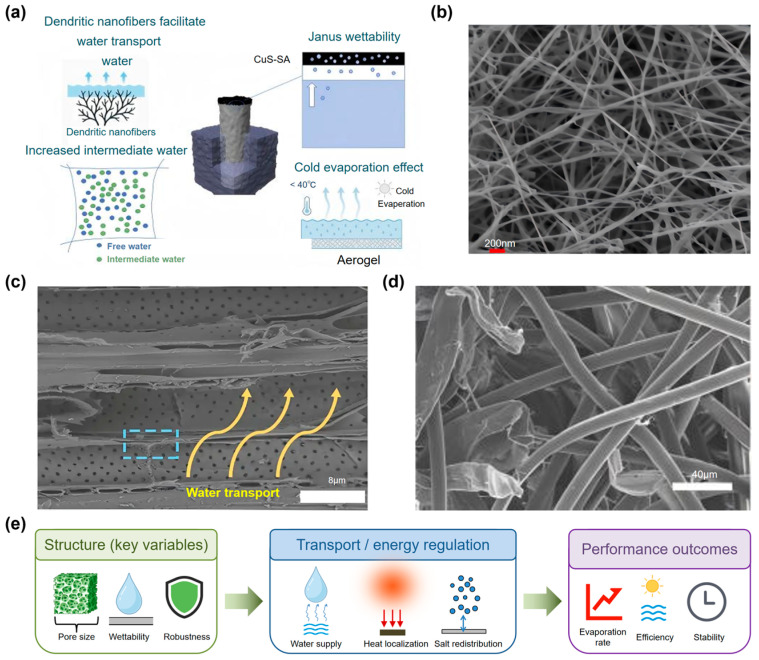
Structure–transport–performance relationship of representative cellulose-based porous substrates for interfacial solar steam generation (ISSG). (**a**) CNF-based aerogel showing aligned pores, Janus wettability, and crosslinked fibrillar networks. Adapted from ref. [90] with permission from Elsevier, © 2025 Elsevier Ltd. (**b**) Cross-sectional morphology of LN_2_ pre-freezing bacterial cellulose aerogel (BCA), showing a wet-stable biosynthesized nanofiber network. Reproduced from ref. [132] under the Creative Commons Attribution (CC BY) license, © 2025 by the authors. (**c**) Micromorphology of DW–TA–Fe^3+^, illustrating natural wood-derived axial channels for water transport and vapor release. Reproduced from ref. [133] under the Creative Commons Attribution 4.0 International License. (**d**) SEM image of carbonized air-laid paper (CAP), showing a random interwoven fiber network in a standardized planar substrate. Reproduced from ref. [128] under the Creative Commons Attribution (CC BY) license, © 2018 Lin et al. (**e**) Schematic structure–transport–performance map linking pore size, connectivity, orientation, wettability, and robustness to water supply, vapor release, heat localization, salt redistribution, evaporation performance, salt resistance, stability, and module readiness. Panel (**e**) is original artwork created by the authors.

The choice of porous substrate should follow the logic of specific application. Aerogels with adjustable height are more suitable for mechanical research and high-performance prototypes; wood and cotton fabric are more suitable for expandable evaporator module; and agricultural residues or commercial paper are more suitable for low-cost or replaceable water treatment units. From the deployment point of view, the substrate with precise structure usually provides better transportation adjustment but its manufacturing readiness is low, while the naturally derived or commercial cellulose substrate provides stronger compatibility for scalable processing, replacement and module-level integration. In order to summarize the design logic related to the structure of cellulose substrate, Figure 3 compares the representative substrate structures, and illustrates how pore size, connectivity, orientation, wettability and structural robustness jointly control water supply, steam release, thermal localization, salt redistribution and final equipment performance.

## 4. Multifunctional Design Strategies for Cellulose-Based ISSG Substrates

**Figure 4:**
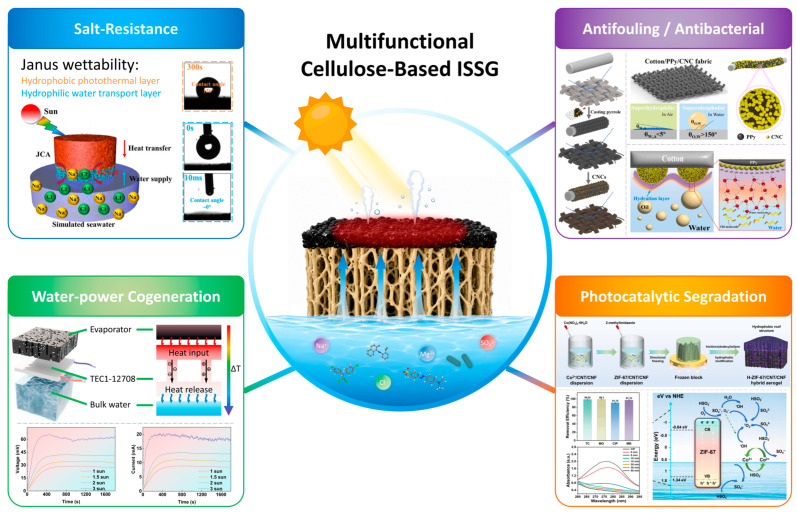
Four typical multifunctional design strategies of cellulose-based interfacial solar steam generation (ISSG), including salt resistance, antifouling/antibacterial design, water–electricity cogeneration, and photocatalytic degradation. Adapted from ref. [91] with permission from Elsevier, © 2025 Elsevier Ltd. All rights reserved.

Under simplified laboratory conditions, ISSG substrates based on cellulose usually show good evaporation performance, but the real water environment will exert coupling stress, which cannot be solved only by evaporation rate. Salt crystallization, organic fouling, microbial growth, limited energy recovery and persistent pollutants can block transmission paths, change interfacial wettability, weaken photothermal conversion and reduce long-term stability. Equipment design has changed from single-function evaporation to integrated operation. The main strategies include salt regulation, antifouling and antibacterial interface, hydropower co-production and photocatalytic degradation (Figure 4). These functions are not independent additional functions, and they must be coordinated with water transport, thermal localization, pore stability and interface chemistry to maintain performance under actual operating conditions. Multifunctional cellulose evaporators should be judged by function compatibility, not by the number of integrated features. Salt resistance, antifouling, power generation and photocatalysis can improve practical value, but each function may also compete with evaporation through hole plugging, increased thermal conductivity, decreased wettability control, catalyst leaching or higher device complexity. In order to avoid describing versatility as a simple additional concept, Table 7 summarizes the technical logic and main trade-offs of representative multifunctional strategies.

### 4.1. Salt-Resistance Design

Salt crystallization is still the main reason for the performance degradation of ISSG system. In the evaporation process, with the removal of water, dissolved ions gather near the air–water interface. Once the local supersaturation is reached, the crystals nucleate and grow on the photothermal surface or in the transport channel. Salt deposition reduces light absorption, limits steam release, blocks water channels, and can deform porous structures. Therefore, salt tolerance depends on the balance between evaporation rate, capillary replenishment, ion back diffusion, nucleation behavior and mechanical tolerance of substrate.

Current salt-resistant designs can be organized into passive exclusion, directional redistribution, interfacial crystallization control, structural reinforcement, and adaptive regulation. Janus architectures are the most common passive strategy [70,138,139,140]. A hydrophilic lower layer maintains water supply, whereas a hydrophobic or partially hydrophobic upper layer limits brine retention near the photothermal region and shifts crystallization away from the active evaporation surface. This design is effective under moderate salinity, but its performance can decline during prolonged operation or under highly concentrated brine because salt transport and crystal removal remain limited.

Directional transport structures provide a more active route by controlling where ions migrate and where salt crystallizes. Inspired by plant vascular systems, cellulose aerogels with coupled vertical and radial channels use capillary flow and concentration gradients to guide concentrated brine from the evaporation center toward peripheral regions [141,142,143,144]. The goal is not to eliminate crystallization but to relocate salt deposits away from active transport pathways. This strategy is particularly relevant for continuous operation, where complete salt prevention is difficult.

Interfacial chemistry regulates salt adhesion and crystal morphology. Properly designed surfaces can induce loose and weakly attached crystals rather than dense salt crusts, making salt removal easier through gravity, rinsing, or flowing water [145]. Molecular regulation offers another level of control. Enhanced hydration within cellulose-based matrices can promote ion mobility, delay local supersaturation, and reduce crystallization in confined channels [146,147,148]. These mechanisms show that salt management depends not only on wettability, but also on the coupling between ion transport, water state, and nucleation kinetics.

Mechanical stability is also essential because salt growth exerts stress on porous frameworks. Reinforcing the cellulose network with nanomaterials or stronger interfacial bonding can improve resistance to crystallization-induced deformation and preserve pore continuity during repeated salt deposition and removal [141,149]. Without mechanical resilience, even an interface with good salt rejection may fail after multiple scaling cycles.

Responsive interfaces represent a more advanced form of salt regulation. Photoresponsive or pH-responsive materials can adjust surface wettability according to external conditions [145,150,151]. Under strong irradiation, a more hydrophobic surface can promote brine discharge and reduce salt retention. Under weak irradiation or dark conditions, recovered hydrophilicity can restore water replenishment. This adaptive behavior shifts salt management from fixed structural protection to dynamic regulation, which is important for high salinity water and fluctuating outdoor environments.

Despite these advances, salt-resistant cellulose-based evaporators still require more realistic evaluation. Many studies demonstrate salt tolerance over short durations or fixed salinity, whereas practical operation involves fluctuating sunlight, variable brine concentration, repeated drying wetting cycles, and mixed foulants. Future designs should report salt accumulation rate, crystal location, cleaning recovery, pore retention, and evaporation stability over repeated cycles.

Salt-resistant design must balance ion removal and water supply. Increasing hydrophobicity can reduce brine retention near the hot surface, but excessive hydrophobicity can slow capillary replenishment. Enlarged channels promote ion back diffusion, but they may reduce thermal confinement. Dense networks improve mechanical resistance to salt growth, but they may restrict vapor release. These trade-offs explain why short-term salt rejection does not always translate into long-term desalination stability. While enhancing salt rejection improves long-term evaporation stability, dense coatings or high additive loading may reduce water flux and limit thermal localization, requiring careful balance.

### 4.2. Antifouling and Antibacterial Design

Biofouling in cellulose-based ISSG systems usually begins with microbial adhesion, followed by colonization, extracellular polymer secretion, and biofilm formation. Established biofilms cover the evaporation interface, reduce light absorption, hinder interfacial heat transfer, block water pathways, and may accelerate cellulose degradation through microbial enzymes. Antifouling and antibacterial design must therefore prevent initial adhesion, suppress microbial growth, and preserve water transport and photothermal conversion during long-term operation in natural water or wastewater.

Physical anti-adhesion strategies reduce contact between microorganisms and the evaporator surface. Hydrophobic modification, including alkyl chain grafting and fluorinated silane treatment, creates low-adhesion interfaces that limit bacterial attachment by reducing solid–liquid contact [152,153]. Liquid-infused porous surfaces follow a similar principle by maintaining a lubricating layer that inhibits adhesion and assists contaminant removal. These strategies can reduce early-stage fouling, but their durability depends on coating stability, abrasion resistance, and retention of surface chemistry during wet operation.

Chemical antibacterial strategies rely on active species that damage or inhibit microorganisms after contact. Silver nanoparticles, copper nanoparticles, and related antimicrobial agents can be generated in situ or loaded into cellulose-based matrices. Released metal ions disrupt microbial membranes and interfere with cellular metabolism [141,150]. Oxidizing surface hydroxyl groups to aldehyde groups provides a metal-free route, giving cellulose frameworks intrinsic antibacterial activity while reducing dependence on leachable inorganic agents [154]. The main challenge is to balance antibacterial efficacy with ion release control, material safety, and long-term structural integrity.

Active self-cleaning designs use the operating process of ISSG to renew the evaporation interface. Photothermal layers generate localized heat under irradiation, enabling intermittent thermal sterilization and weakening biofilm adhesion [155]. Vertically aligned channels can promote vapor-induced microconvection and help remove loosely attached cells or organic deposits [156]. Compared with static anti-adhesion coatings, these designs use evaporation, heating, and flow to limit fouling accumulation, which is more relevant for bioactive water streams.

Integrated antifouling systems combine multiple mechanisms within one cellulose-based architecture. Photothermal materials and antibacterial nanoparticles, such as polypyrrole and silver nanoparticles, can be incorporated into cellulose networks to couple solar heating with bacterial inactivation [157,158]. Janus architectures provide another route by spatially separating water-facing and air-facing functions. The lower region maintains water uptake, while the upper region combines antibacterial activity, photothermal conversion, and antifouling wettability [159]. Such integration better reflects practical conditions, where salt deposition, organic matter accumulation, microbial growth, and thermal cycling often occur together.

The central issue for future antifouling design is durability rather than initial antibacterial efficiency. Effective systems should retain antibacterial activity after repeated wetting, drying, salt exposure, cleaning, and mechanical disturbance. They should also minimize leaching of antimicrobial agents and avoid blocking water pathways. For cellulose-based ISSG to operate in natural water and wastewater, antifouling design must evolve from antibacterial loading toward renewable, low-leaching, and transport-preserving interfaces.

Antifouling design also involves competing requirements. Hydrophobic coatings reduce microbial adhesion, but they may reduce water uptake. Antibacterial nanoparticles improve sterilization, but ion leaching raises durability and safety concerns. Dense protective layers improve fouling resistance, but they may block vapor pathways. A useful antifouling cellulose evaporator should maintain water transport and photothermal conversion after repeated fouling and cleaning cycles. Antibacterial or antifouling coatings can prevent biofouling, but excessive agent concentration may lead to leaching, reduce biodegradability, or alter pore structure, thereby affecting evaporation efficiency.

### 4.3. Water and Electricity Cogeneration

Hydropower cogeneration expands cellulose-based ISSG from water production to simultaneous water and energy recovery. Two mechanisms are mainly involved. Evaporation-induced power generation relies on continuous water flow and evaporation-driven ion migration to create electrokinetic output. Thermoelectric conversion uses temperature gradients generated by photothermal evaporation or waste heat dissipation to produce electricity. Both mechanisms aim to recover part of the energy that is otherwise lost during solar desalination.

Material-integrated systems embed evaporation and power generation within the same membrane or aerogel. Hybrid-dimensional membranes combining two-dimensional reduced graphene oxide with one-dimensional chitin fiber carbon nanotube structures create layered nanochannels for photothermal evaporation and selective ion migration [160,161,162]. In these systems, channel size, surface charge, and functional groups regulate ion transport during evaporation, allowing for electrical output without an external energy module. Bionic cellulose graphene aerogels with radial channels provide another material level strategy by mimicking tree transpiration or cork like radial transport, thereby coupling water flow, interfacial evaporation, and evaporation-induced charge separation [163,164].

System-integrated devices couple cellulose-based evaporators with photovoltaic or thermoelectric modules. In photovoltaic integrated systems, the evaporator works as both a freshwater generator and a cooling layer. CNF-based bilayer aerogels with carbon nanotube photothermal layers can reduce the operating temperature of the underlying photovoltaic cell through evaporative cooling, improving photoelectric conversion while maintaining water production [165,166]. In thermoelectric systems, the temperature difference generated during solar evaporation drives a thermoelectric module, converting part of the thermal gradient into electrical output [118,167]. In this configuration, unavoidable heat dissipation is converted into auxiliary energy.

These two routes differ in device logic. Material-integrated systems offer compact architectures and use ion transport within the evaporator itself, but their electrical output is often limited by ion concentration, channel stability, humidity, internal resistance, and interfacial charge density. System-integrated designs generally provide more controllable power output through mature photovoltaic or thermoelectric components, but they increase device complexity and require careful thermal and structural matching. Improper coupling may reduce evaporation performance by disturbing heat localization or water supply.

For water and electricity cogeneration to become practical, the key issue is not whether electricity can be generated, but whether energy recovery improves the overall system value without compromising water production. Conductive fillers and thermoelectric modules can increase electrical output, but they may increase heat leakage, block pores, raise internal resistance, or complicate module assembly. Future studies should report water yield and electrical output under the same operating conditions, together with internal resistance, thermal balance, and stability during continuous evaporation. Integration of electricity generation enhances energy recovery but may increase thermal conductivity or structural rigidity, potentially reducing water evaporation efficiency and mechanical flexibility.

### 4.4. Photocatalytic Degradation

Photocatalytic degradation extends cellulose-based ISSG from physical water separation to combined evaporation and chemical oxidation. In these systems, photothermal components drive interfacial evaporation, while photocatalysts activated by solar irradiation generate reactive oxygen species that degrade dissolved organic pollutants into less harmful or mineralized products [168]. This coupling is relevant for wastewater-containing dyes, antibiotics, volatile organic compounds, or other persistent contaminants, because evaporation alone cannot remove nonvolatile pollutants and may transfer volatile pollutants into the condensate.

The coupling between photothermal evaporation and photocatalysis arises from shared solar input and interfacial process enhancement. Localized heating accelerates water transport, enriches pollutants near the reaction interface, and increases contact between pollutants and catalytic sites. Under illumination, photocatalysts generate oxidative species that decompose refractory organics. Manganese ferrite, metal–organic frameworks, manganese dioxide, TiO_2_-based composites, ferrites, and related catalytic components have therefore been incorporated into cellulose-based matrices to combine light absorption, heat generation, and advanced oxidation [168].

Photocatalytic cellulose evaporators can be designed through direct catalyst loading, waste-derived catalyst integration, or multicomponent coupling. Direct loading places catalytic nanoparticles onto cellulose substrates. Manganese iron nanoparticles prepared by coprecipitation, for example, enable interfacial evaporation, dye degradation, and hydrovoltaic power generation in one membrane [169]. Multicomponent composites integrate several catalytic and photothermal pathways, as shown by reduced graphene oxide, TiO_2_, nickel ferrite, and zinc oxide systems for evaporation and methylene blue degradation [170].

Recent designs have extended photocatalytic ISSG toward electrochemically assisted oxidation. Coupling photothermal evaporation with electro-Fenton chemistry allows electrocatalytic components to generate reactive oxygen species in situ and degrade volatile organic pollutants in condensed water [171]. By coupling evaporation with electro-Fenton oxidation, this design addresses a limitation of conventional evaporation systems, where volatile contaminants may transfer from source water to condensate rather than being retained or destroyed. It shifts the design target from separation alone to coupled separation and degradation.

The main challenge is to match evaporation kinetics with oxidation kinetics. Excessive catalyst loading can block water pathways, increase heat loss, reduce mechanical integrity, and weaken vapor release. Insufficient loading limits pollutant degradation. Catalyst stability, radical generation, reaction selectivity, byproduct formation, and condensate quality should be evaluated under realistic water compositions. Photocatalytic cellulose evaporators should therefore be judged by both water yield and contaminant removal efficiency, rather than by evaporation rate alone. Photocatalyst incorporation facilitates pollutant degradation, yet high catalyst loading may block pores or increase heat dissipation, limiting local temperature rise and water flux.

## 5. Summary, Challenges, and Future Perspectives

**Figure 5:**
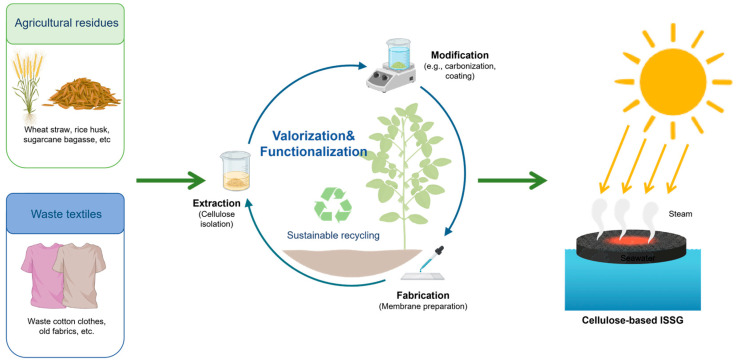
Schematic representation of the “waste-to-value” strategy for transforming agricultural residues and waste textiles into high-performance cellulose-based solar steam generators.

This review has examined cellulose-based ISSG substrates through a structure-guided perspective. Cellulose contributes to ISSG performance through hydroxyl-mediated hydration, fibrillar network formation, hierarchical porosity, low thermal conductivity, and chemical modifiability. These features control water transport, thermal localization, salt redistribution, mechanical stability, and multifunctional integration. Different cellulose platforms show distinct advantages. CNF aerogels provide structural tunability. BC networks provide wet stability. Wood provides aligned channels. Cotton fabrics provide flexible processing. Agricultural residues provide low-cost feedstocks. Commercial cellulose paper provides standardization and replaceability (Figure 5). Cellulose-based ISSG remains an emerging distributed water-treatment platform. It is not yet a commercially mature desalination technology.

### 5.1. Key Bottlenecks for Practical Deployment

The translation of cellulose-based ISSG from laboratory demonstrations to practical deployment remains constrained by several unresolved bottlenecks. First, scalable manufacturing is still limited. Many high-performance evaporators rely on directional freezing, freeze-drying, supercritical drying, or multistep nanomaterial assembly. These methods are useful for precise pore regulation, but they increase process complexity, energy consumption, and production cost. Scalable manufacturing should prioritize ambient drying, roll-to-roll coating, textile finishing, and papermaking over freeze-drying and supercritical drying. The latter should be reserved for mechanism-driven research until their energy costs decrease.

Second, long-term operational stability remains insufficiently validated. Many studies have reported the short-term evaporation performance under simplified laboratory conditions, but the actual operation involves fluctuating sunlight, wind, humidity, water temperature, salinity, fouling pressure and mechanical disturbance. Under repeated wetting–drying cycles, cellulose-based evaporators may suffer from coating delamination, pore collapse, salt crystallization, microbial growth, swelling and aging. Therefore, future research should evaluate evaporation stability, salt-cleaning recovery rate, pollution resistance, mechanical integrity and photothermal layer adhesion under actual outdoor conditions.

Thirdly, salt management is still the main challenge of continuous desalination. Passive salt tolerance is usually effective under moderate salinity or short-term operation, but its performance may decrease under concentrated salt water, intermittent sunlight or repeated drying. The future design should shift from static salt exclusion to dynamic salt regulation. Responsive wettability, guided ion transport, local crystallization and self-cleaning structure can help control where salt nucleates and how it is removed.

Fourthly, standalone solar evaporation is limited by the intermittent solar energy and the inherent upper limit of photothermal water evaporation. Multi-energy coupling can extend operation beyond ideal daytime conditions. Possible approaches include electrothermal resistance, thermoelectric recovery, photovoltaic cooling, waste heat utilization and solar chemical coupling. However, additional energy modules should not be evaluated only by functional diversity. It must be evaluated according to its net contribution to water production, energy recovery, system complexity and maintenance cost.

Fifth, multi-functional integration often involves trade-offs. Salt resistance, pollution prevention, power generation and photocatalytic degradation can improve practical value, but they may also compete with evaporation. Photocatalyst loading can block the vapor pathway. Antimicrobial agents may leach during operation. Conductive fillers will increase heat leakage. A dense protective coating can reduce the supply of capillary water. Therefore, multifunctional cellulose ISSG should be evaluated by functional compatibility, not by the number of functions integrated into a device.

### 5.2. Practical Translation and Commercialization Readiness

Most cellulose-based ISSG systems are still at the material-prototype stage rather than the deployable module stage. Practical translation depends not only on evaporation rate and solar-to-vapor efficiency, but also on manufacturing route, material cost, process energy demand, outdoor durability, replacement feasibility, condensation efficiency, and module-level water collection. To make the commercialization discussion more explicit, an indicative technology-readiness-level (TRL)-style assessment is introduced here (Table 8). Because most cellulose-based ISSG studies remain at the laboratory or material-prototype stage, the assigned TRL values should not be interpreted as formal industrial certification. Instead, they provide a compact comparative framework that links representative quantitative performance, fabrication scalability, durability evidence, module compatibility, and remaining deployment gaps. Where available, reported evaporation rates, solar-to-vapor efficiencies, outdoor tests, salinity tests, and operation durations are used as quantitative anchors. Where such values are not consistently reported, the absence of standardized data is identified as a key barrier to techno-economic evaluation.

CNF and BC aerogels are valuable for the study of oriented mechanism because their pore orientation, wettability and composition distribution can be designed with high precision. However, their practicability is limited by drying energy, shrinkage, batch uniformity and large-scale manufacturing. Wood and cotton fabrics are closer to expandable modular structures because their natural channels or textile networks have provided water transmission paths. Agricultural residues and commercial cellulose paper provide stronger cost advantages and easier replacement, but their performance reproducibility and long-term stability need stricter quality control.

The manufacturing strategy determines commercialization potential. Freeze-drying and directional freezing offer structural control but limit scale-up due to energy cost and batch processing. Coating, roll-to-roll processing, textile finishing, and ambient drying are compatible with continuous production and should be prioritized for module-scale manufacturing. In contrast, coating, printing, papermaking, reel-to-reel processing, textile finishing and normal temperature drying are more suitable for continuous production. Therefore, future research should report not only evaporation performance, but also material load, manufacturing energy, output, processing time and potential area expansibility.

Module-level design is another missing link between laboratory performance and real deployment. Practical ISSG devices must include not only evaporators, but also water supply, steam transportation, condensation, salt cleaning, mechanical support and maintenance strategies. In many laboratory studies, evaporation is measured from the open surface, but the efficiency of fresh water collection is not fully quantified. Freshwater cost cannot be estimated without standardized techno-economic reporting. Future studies should report standardized module-level and techno-economic indicators, including absorber cost per unit area, fabrication energy, membrane lifetime, daily outdoor water yield, condensation efficiency, cleaning frequency, replacement interval, and performance recovery after long-term operation. Without these parameters, meaningful cost comparisons with conventional desalination remain impossible. Its near-term applications lie in decentralized purification, emergency supply, small-scale desalination, off-grid communities, and low-cost pretreatment—not in large municipal plants.

The TRL-style assessment suggests that commercialization readiness is not determined by evaporation performance alone. Although aerogel-based systems often achieve strong laboratory performance, their scalability remains limited by energy-intensive fabrication and manufacturing complexity. In contrast, wood-, textile-, and paper-based platforms currently offer a more practical pathway toward module-scale deployment because they are compatible with natural channels, textile processing, or papermaking routes. Future studies should complement evaporation-rate reporting with standardized indicators such as fabrication energy, material cost, operational lifetime, outdoor water yield, condensation efficiency, replacement interval, and end-of-life treatment.

Beyond commercialization readiness, sustainability also requires cautious evaluation. Cellulose-based ISSG systems should not be assumed to be inherently low-carbon or fully circular. Their environmental footprint depends on processing energy, drying or carbonization route, functional additive loading, device lifetime, and end-of-life management. Potential leaching or persistence of Ag, MXene, TiO_2_, CNTs, graphene derivatives, and other additives should also be considered when evaluating environmental safety.

### 5.3. Future Directions

Looking ahead, cellulose-based ISSG may evolve through three stages. The first generation is represented by single-function evaporators, which are mainly used for solar desalination and fresh water production. These systems focus on photothermal conversion, water transport and heat localization. The second generation is becoming a multifunctional platform, which combines evaporation with salt resistance, anti-pollution behavior, power generation or pollutant degradation. These systems expand the role of ISSG from simple water production to water–energy–environment coupling application.

The third generation, which has just begun to take shape, should be intelligent, adaptive and integrated. This system should respond to fluctuating salinity, sunlight, humidity, fouling pressure and water composition by dynamically adjusting transport pathway, interface chemistry and energy distribution. Possible design directions include switchable wettability, adaptive salt crystallization control, self-cleaning interface, responsive photothermal layer, modular water collection unit and multi-energy coupling. These strategies can make the cellulose-based ISSG work more reliably under non-ideal outdoor conditions.

Future development should also shift from performance maximization to performance reliability. The high evaporation rate under one-sun laboratory conditions should not be regarded as the only indicator of progress. More information standards include continuous water production, condensate quality, scaling recovery, salt tolerance duration, membrane life, replacement cost and environmental safety. Standardized testing protocols are particularly important for comparing different cellulose platforms and determining which systems are suitable for practical deployment.

In this broader context, cellulose-based ISSG is not only a material topic or a desalination strategy. Cellulose-based substrates may provide a promising route toward distributed and resource-efficient water treatment, provided that scalable manufacturing, outdoor durability, module integration, environmental safety, end-of-life management, and cost validation are systematically addressed.

## Figures and Tables

**Table 1:** Comparison of existing cellulose-based ISSG reviews and the present work based on structural, functional, and practical perspectives.

Existing Reviews	Structural Hierarchy Analysis	Structure–Function Correlation	Multifunctionality	Commercialization	Sustainability	Future Roadmap	Reference
Biomass-derived ISSG reviews	Partial	Limited	Limited	None	None	Limited	[23,31,32]
Nanocellulose ISSG reviews	Moderate	Moderate	Limited	None	None	Limited	[33,34,35,36]
Wood-based ISSG reviews	Moderate	Limited	Limited	None	None	Limited	[37,38,39,40]
Multifunctional ISSG reviews	Limited	Limited	Yes	Limited	None	Limited	[41,42,43]
This Review	Molecular → Macro-scale	Cross-length-scale functional analysis	Salt, antifouling, electricity, photocatalysis	Technology readiness level (TRL) and scalable fabrication	Energy, materials, waste-to-value	3-generation ISSG, adaptive strategies	-

**Table 7:** Technical logic and trade-offs of multifunctional cellulose-based ISSG systems.

Multifunctional Strategy	Technical Logic	Main Trade-Off	Design Implication	Reference
Salt resistance	Regulate ion transport and salt crystallization.	Salt rejection ↔ water flux	Maintain salt removal without blocking water supply	[134]
Antifouling/antibacterial design	Suppress adhesion, biofilm formation, and contamination.	Antibacterial activity ↔ leaching risk	Use stable, low-leaching antibacterial components	[135]
Water–electricity cogeneration	Harvest thermal gradients or evaporation-driven ion migration.	Power output ↔ heat localization	Control conductive pathways to avoid heat loss	[136]
Photocatalytic degradation	Couple evaporation with pollutant oxidation.	Catalyst loading ↔ vapor transport	Optimize catalyst amount to avoid pore blockage	[137]

Note: Multifunctional design should prioritize function compatibility rather than simply increasing the number of integrated functions.

**Table 8:** Compact TRL-style assessment of cellulose-based ISSG platforms.

Platform	Quantitative Anchor	Scale-Up Potential	Main Bottleneck	Indicative TRL	Reference
BC aerogels/hydrogels	~1.13–3.43 kg m^−2^ h^−1^	Medium	Biosynthesis uniformity; drying shrinkage	3–4	[70]
CNF aerogels	~1.97–3.20 kg m^−2^ h^−1^	Medium	Freeze-drying, shrinkage, cost	3–4	[172]
Wood substrates	~1.42–3.40 kg m^−2^ h^−1^	High	Natural variability; coating durability	4–5	[173]
Cotton fabrics	~1.39–3.16 kg m^−2^ h^−1^	High	Washing, bending, coating adhesion	4–5	[174]
Agricultural residues	Variable; low-cost feedstocks	Medium	Feedstock variability, reproducibility	3–4	[175]
Cellulose paper	~0.96–1.97 kg m^−2^ h^−1^	High	Moderate performance, weak anisotropy	4–5	[176]
Multifunctional hybrids	Water–energy–pollutant coupling	Low–Medium	Complex integration, stability	2–3	[177]

Note: The TRL values are indicative rather than formal industrial ratings. They are assigned based on reported performance, fabrication scalability, durability evidence, and module compatibility. Direct performance comparison should be made cautiously because testing conditions differ among studies.

## Data Availability

The original contributions presented in this study are included in the article. Further inquiries can be directed to the corresponding author.
